# Triticale doubled haploid plant regeneration factors linked by structural equation modeling

**DOI:** 10.1007/s13353-022-00719-7

**Published:** 2022-08-26

**Authors:** Renata Orłowska

**Affiliations:** Plant Breeding and Acclimatization Institute–National Research Institute, Błonie, Radzików, 05-870 Poland

**Keywords:** Androgenesis, DNA methylation, Green plant regeneration efficiency, Sequence variation, Triticale

## Abstract

Triticale regeneration via anther culture faces many difficulties, e.g., a low percentage of regenerated plants and the presence of albinos. Plant regeneration may be affected by abiotic stresses and by ingredients added to the induction medium. The latter influences biochemical pathways and plant regeneration efficiency. Among such ingredients, copper and silver ions acting as cofactors for enzymatic reactions are of interest. However, their role in plant tissue cultures and relationships with biochemical pathways has not been studied yet.

The study evaluated relationships between DNA methylation, changes in DNA sequence variation, and green plant regeneration efficiency influenced by copper and silver ions during triticale plant regeneration. For this purpose, a biological model based on donor plants and their regenerants, a methylation-sensitive amplified fragment length polymorphism, and structural equation modeling were employed.

The green plant regeneration efficiency varied from 0.71 to 6.06 green plants per 100 plated anthers. The values for the components of tissue culture-induced variation related to cytosine methylation in a CHH sequence context (where H is A, C, or T) were 8.65% for sequence variation, 0.76% for DNA demethylation, and 0.58% for de novo methylation. The proposed model states that copper ions affect the regeneration efficiency through cytosine methylation and may induce mutations through, e.g., oxidative processes, which may interfere with the green plant regeneration efficiency. The linear regression confirms that the plant regeneration efficiency rises with increasing copper ion concentration in the absence of Ag ions in the induction medium. The least absolute shrinkage and selection operator regression shows that de novo methylation, demethylation, and copper ions may be involved in the green plant regeneration efficiency. According to structural equation modeling, copper ions play a central role in the model determining the regeneration efficiency.

**Supplementary Information:**

The online version contains supplementary material available at 10.1007/s13353-022-00719-7.

## Introduction

Green plant regeneration efficiency (GPRE) via anther culture of breeding species is a key prerequisite for developing new varieties. Numerous efforts were undertaken to overcome the low GPRE (Oleszczuk et al. 2004; Ślusarkiewicz-Jarzina et al. 2017). Among others, stresses such as cold (Tenhola-Roininen et al. 2006), heat (Li et al. 2016), darkness (Żur et al. 2019), starvation (Touraev et al. 1996), or their combinations (Zhuang and Xu 1983) were tested with varying success. The mechanisms of stress action in plant androgenesis are still extensively studied (Hale et al. 2020). The same is also true in the case of the biochemical background of the stresses (Zieliński et al. 2021). However, the biochemical background of the GPRE could be tuned via addition to the induction medium (IM) of key compounds that might work as cofactors of enzymatic reactions participating in the cellular pathways (Camponeschi and Banci 2019; Castillo-González et al. 2018; Schmidt and Husted 2019). Among others, metal ions (i.e., copper–Cu(II), zinc–Zn(II), silver–Ag(I)) added to the IM may influence many cellular processes, resulting in complex interactions, possibly affecting GPRE.

Cu(II) ions are cofactors that may affect the electron transport chain (ETC), which produces ATP through its five complexes embedded in the inner membrane (Mansilla et al. 2018 5595). Copper is present in a complex IV called cytochrome c oxidase (COX). Complex IV participates in electron transfer (Mansilla et al. 2018) and is in charge of cellular energy production via complex V (Bailleul et al. 2015; Seelert and Dencher 2011). COX disruption may limit the supply of ATP from the respiratory chain, preventing development beyond the embryo stage in Arabidopsis (Dahan et al. 2014). Hence, Cu(II) is necessary for the proper functioning of the Yang cycle, which is dependent on the energy source of ATP and methionine, resulting in S-adenosyl-L-methionine (SAM) (Pattyn et al. 2021). SAM is involved in the conversion of homocysteine to cysteine (the transsulfuration pathway), which is a precursor of glutathione (GSH) (Lu 2000). Finally, SAM is a primary donor of methyl moieties for the methylation of various compounds in transmethylation reactions (Chiang et al. 1996), including cytosine residues (Van de Poel et al. 2013) and may impact microspore reprogramming from gametophytic to sporophytic fate (Maraschin et al. 2005) via methylation-dependent regulation of gene expression. Thus, stress-induced microspore embryogenesis requires fine-tuning of many cellular processes (Testillano et al. 2000).

About 1% of the oxygen consumed by plants is converted to reactive oxygen species (ROS) during cell cycles and at varying subcellular locations (Bhattacharjee 2005). Copper- and zinc-containing superoxide dismutase (Cu/Zn-SOD) manages the ROS level and is relevant to the oxidative safety of the cell (Alscher et al. 2002; Kliebenstein et al. 1998). ROS production is associated with plant exposure to abiotic and biotic stresses in plants. Hence*,* in vitro culture conditions, which are inherently stressful, also drive ROS production, impacting the formation of in vitro-induced variation (Cassells and Curry 2001; Krishna et al. 2016). ROS activity may also affect DNA methylation through the oxidation of 5-methylcytosine (5mC) (El-Maarouf-Bouteau et al. 2011; Kurek et al. 2019; Michalak et al. 2015) resulting in DNA demethylation via repair pathways involving base excision. However, not all cytosine modifications are easily removed, and some may lead to mutations. The deamination process of 5mC results in thymine (T) that generates the C → T transition (Bellacosa and Drohat 2015). Thus, oxidative stress in plant tissue cultures may prime changes in DNA methylation (Moricová et al. 2013) and influence DNA sequence variation in the form of point mutations (deletions, substitutions) (Neelakandan and Wang 2012).

The well-functioning mechanism encompassing ROS-scavengers, including Cu/Zn-SOD, might limit DNA sequence changes. But under in vitro tissue culture conditions, the action of Cu/Zn-SOD can be accentuated by other metal ions such as silver. In the Cu/Zn-SOD, silver can substitute for the site occupied by copper, forming an Ag/Zn-SOD variant. When AgNO_3_ was added to the growth medium for *Saccharomyces* *cerevisiae*, the Cu/Zn-SOD activity dropped by a factor of 6 (Ciriolo et al. 1994). ROS production may also be controlled by melatonin (N-acetyl-5-methoxytryptamine) (Hardeland 2016) produced in chloroplasts and mitochondria (Tan et al. 2013). The ability of melatonin to donate an electron or hydrogen atom animates free radical detoxification and reduces oxidative stress (Galano et al. 2018). Melatonin raises glutathione levels, chelating excess Cu(II) (Galano et al. 2015). In vivo, melatonin protects against Cu(II) (via a copper-chelating agent rather than a hydroxyl scavenger mechanism).

Despite Cu/Zn-SOD, there may be another crossover between the copper and silver ions in plant tissue cultures due to similar physicochemical and electronic properties of Cu(II) and Ag(I) (Roe et al. 1990) allowing copper substitution by silver, i.e., in ethylene receptor (ETR1). Copper, as an essential element of the ETR1, acts as a cofactor in the trans-membrane part of the receptor (Rodríguez et al. 1999). Thus, the presence of copper allows gaseous ethylene molecules to attach and initiate varying cellular processes. In general, ethylene plays an essential role in the regeneration of plants in vitro (Kumar et al. 2009). In addition, silver ions may interact with the ETR1 receptor and partially hinder the binding of ethylene to the receptor, inhibiting responses to ethylene when applied in vitro (Jha et al. 2007).

Together, Cu(II) in the IM appears to be a significant component regulating the transcriptome, cell reprogramming phases, and metabolic pathways that may impact GPRE (Bednarek and Orłowska 2020). According to the evidence that was available, increased Cu(II) concentration improved GPRE in barley and other culture (Dahleen 1995; Jacquard et al. 2009). On the other hand, mediation studies in a different culture of triticale did not indicate the beneficial effects of Ag(I) ions in the IM. At the same time, the role of Cu(II) was evident (Orłowska et al. 2022). For GPRE in some species, Cu(II) and Ag(I) both appear to be necessary. However, it is unclear how the ions cooperated in the tissue culture media to raise GPRE.

DNA methylation is one of the three elements responsible for epigenetic mechanisms. In plants, DNA-methylated cytosine appears in two types of sequence contexts: symmetric (CG, CHG) and asymmetric (CHH) (H is A, C, or T) (Cokus et al. 2008; Lister et al. 2008). The distribution of methylated cytosine varies across sequence contexts (Feng et al. 2010) and genome regions (Hua et al. 2020) and fluctuates during biological processes (Li et al. 2019). Similarly, there is variability regarding methylation in each context depending on the tissue studied, so in the rapeseed genome, the most significant differences were identified between shoots and roots in the context of CHH (Hua et al. 2020). As shown by the levels of gene expression and CHH methylation in the promoter regions of *Populus* *tomentosa* (Chen et al. 2021), methylation of CHH may be an important part of some cellular processes, such as plant hormone signal transduction, starch and sucrose metabolism, and phenylpropanoid biosynthesis. Also, changes in DNA methylation in the CHH context can be attributed to genes encoding a copper-binding family protein (Chen et al. 2021). Other studies showed that changes in DNA methylation are mainly contributed by the presence of 5mC in CHH sequences (Chen et al. 2020; Ji et al. 2019). Because of this, the above evidence about the CHH context predisposes it to detailed analysis in studies on methylation changes in triticale in vitro cultures.

The study of tissue culture-induced variation (TCIV) should be carried out in a specially designed biological model (Machczyńska et al. 2015; Orłowska 2021; Orłowska and Bednarek 2020) using suitable detection methods, based on epigenetic and genetic markers, i.e., methylation sensitive amplification polymorphism (MSAP) (Baranek et al. 2010), methyl-sensitive transposon display (MSTD) (Orłowska et al. 2021a), methylation-sensitive amplified fragment length polymorphism (metAFLP) (Orłowska et al. 2021b), and diversity arrays technology methylation analysis (DArTseqMet) (Bednarek and Orlowska 2020). The biological model should consist of the donor plant, which is the generative progeny of a doubled haploid, and regenerants obtained from anther cultures. With this kind of starting material, changes seen in the regenerants can be linked to the in vitro culture. Different methods can carry out an estimation of tissue culture-induced variation. Still, those based on molecular markers fulfill their role because they are relatively cheap, generate many DNA markers, and, as in the case of metAFLP, get information concerning both sequence changes and DNA methylation. Moreover, the latter method allows studying changes in cytosine localization in different contexts: symmetric (CG and CHG) and asymmetric (CHH) (Bednarek and Orłowska 2020).

Relationships between various factors affecting complex biological systems might be studied using regression analysis such as mediation (Bednarek and Orłowska 2020; Bednarek et al. 2020; Orłowska et al. 2021b) and moderation (Bednarek and Orlowska 2020) or using structural equation modeling (SEM) (Bednarek et al. 2021; Kozak and Kang 2006; Shipley 2002). Since the SEM method is less strict than regression analysis, it may show more complex connections between the factors being studied.

Cu(II) in the IM is thought to have an impact on the ETC, S-adenosyl-L-methionine production, and DNA methylation modifications in both symmetrical and asymmetrical sequence settings. Methylated cytosine residues are vulnerable to mutations brought on by ROS and may be under the control of ROS scavengers (i.e., GSH, melatonin). The activity of the Cu/Zn-SOD limiting sequence and DNA methylation variation may be favored by the addition of Cu(II) to the IM. By substituting copper in the active centers, Ag(I) may have an impact on the ETC and Cu/Zn-SOD. Silver ions may also affect how ethylene behaves. Through metabolic pathways, Cu(II) and Ag(I) may both have an impact on the transcriptome. The intricacy of the Cu(II)/Ag(I)-dependent pathways’ interactions may be important for the GPRE. The work uses anther culture in vitro model, metAFLP data, and SEM to assess potential connections between DNA methylation, SV alterations, and GPRE caused by Cu(II) and Ag(I) in the IM.

## Material and methods

### Plant material and tissue culture conditions

The material consisted of winter triticale (X *Triticosecale* spp. Wittmack ex A. Camus 1927) plants, cultivar T28/2 derived from cv. Presto × cv. Mungis cross provided by Sylwia Oleszczuk (Plant Breeding and Acclimatization Institute-NRI, Radzików, Poland).

The donor plants were prepared as the generative progeny of regenerants derived via triticale anther culture. The prime regenerants were obtained from starting triticale plants grown at 16 °C during the 16-h-day and at 12 °C for 8 h per night. Plants were vernalized for 6 weeks at 4 °C for 8 h a day and 16 h at night. The tillers were sheared at the mid to uninucleate microspores’ developmental stage following cold stress at 4 °C for 20 days. After that, tillers were sterilized by soaking in ethanol for 1 min, followed by soaking in 10% sodium hypochlorite for 20 min. Excised anthers were cultured on a semi-solid induction medium (IM) 190–2 (Zhuang and Xu 1983) [90 g l^−1^ maltose + 438 mg l^−1^ glutamine, + 2 mg l^−1^ 2,4-dichlorophenoxyacetic + 0.5 mg l^−1^ kinetin]. Incubation was carried out at 26 °C in darkness. After 35 days, first calli, embryo-like structures, and embryos were plated onto regeneration 190–2 medium (Zhuang and Xu 1983) [0.5 mg l^−1^ naphthalene acetic acid + 1.5 mg l^−1^ kinetin]. The incubation step was carried out at 26 °C during the 16-h day and 8 h per night. Regenerated green plants were transferred to glass flasks with N6I medium (Chu 1978) [2 mg l^−1^ indole-3-acetic acid] for rooting. The vernalization was carried out as before. After vernalization, plants were grown in greenhouse conditions. The spontaneous diploidization of regenerants was estimated by visual inspection of regenerant morphology (plant growth, leaf shape, color, and width, tillering mode, spike number). Spikes from randomly chosen regenerants were self-pollinated. Plants obtained from regenerant seeds were the generative progeny of doubled haploid (DH) and served as the donor plants for the next step.

The main experiment involved testing the effect of Cu(II) and Ag(I) ions added to IM and the time of incubation anthers on IM in triticale anther cultures. The method of obtaining the regenerants was the same as for obtaining the donor plants, except for varying the composition of the inducing media. Eight treatments (A–H) were performed, differing in the supplementation of CuSO_4_ (0.1, 5, 10 µM) and AgNO_3_ (0, 10, 60 µM) and the incubation time (35, 42, 49 days) of the explants on the IM. The selection of concentrations and number of days was based on literature and own research (Orłowska and Bednarek 2020).The incubation time reflected the period from the plating of anthers on the IM to the transfer of callus, embryo-like structures, and embryos to the regeneration medium, which was the same for all treatments. The number of green regenerants was counted per 100 plated anthers in each treatment. This number is called GPRE.

### DNA extraction and metAFLP analysis

Total genomic DNA was isolated from 100 mg of leaves of 37 regenerants using the Plant DNeasy MiniPrep kit (QIAGEN, Hilden, Germany). DNA concentration was assessed using a spectrophotometer at wavelengths of 260 and 280 nm. The integrity of extracted DNA was checked in a 1.2% agarose gel with ethidium bromide staining. Finally, for metAFLP, 500 ng of genomic DNA was prepared. The metAFLP analysis was performed as described previously (Machczyńska et al. 2014; Orłowska and Bednarek 2020). At 37 °C for 3 h, genomic DNA was restricted with enzyme combinations Acc65I (10 U)/MseI (12 U) and KpnI (10 U)/MseI (12 U) (New England Biolabs, US) and deactivated at 70 °C for 15 min. In a ligation reaction at 20 °C for 12 h with T4 DNA ligase (120 U) (NEB, US), synthetic oligonucleotides called adaptors were joined to the freed DNA fragments (Table 1).

**Table 1:** Oligonucleotides applied for metAFLP in triticale

metAFLP oligomer	Sequence 5′ → 3′
Adaptors	
Ad1 Acc65I	CTC GTA GCA TGC GTA CA
Ad2 Acc65I	GTA CTGTACGCATGCTAC
Ad1 KpnI	CTC GTA GCA TGC GTA CAG TAC
Ad2 KpnI	TGTACGCATGCTAC
Ad1 MseI	TAC TCA GGA CTC ATC
Ad2 MseI	GAC GAT GAG TCC TGA G
Preselective primers	GAT GAG TCC TGA GTA AC
Presel Acc65I/KpnI	GCA TGC GTA CAG TAC C
Presel MseI	GAT GAG TCC TGA GTA AC
Labeled γ^32^P selective oligonucleotides	
CG-GAC	CA TGC GTA CAG TAC CGA C
CG-GCA	CA TGC GTA CAG TAC CGC A
CG-GGC	CA TGC GTA CAG TAC CGG C
CG-TCG	CA TGC GTA CAG TAC CTC G
CHG-AGA	CA TGC GTA CAG TAC CAG A
CHG-AGC	CA TGC GTA CAG TAC CAG C
CHG-AGG	CA TGC GTA CAG TAC CAG G
CHG-ATG	CA TGC GTA CAG TAC CAT G
CHG-TGC	CA TGC GTA CAG TAC CTG C
CHG-TTG	CA TGC GTA CAG TAC CTT G
CHH-ATT	CA TGC GTA CAG TAC CAT T
CHH-TAA	CA TGC GTA CAG TAC CTA A
Selective oligonucleotides	
M-CAC	GAT GAG TCC TGA GTA ACA C
M-CGT	GAT GAG TCC TGA GTA ACG T

The ligation product was diluted 1:3 with water and then used as a template for the pre-selective PCR reaction with pre-selective starters (Table 1) in the presence of 0.5 U of Taq polymerase (Qiagen, Hilden, Germany). The electrophoresis of initial PCR products separated on a 1.2% agarose gel was performed to verify the completeness of the digestion and ligation steps. Next, the product of pre-selective PCR was diluted with water (1:20) and used for a selective amplification step with selective primers (Table 1) and in the presence of 0.0125 U of HotStar DNA polymerase (Qiagen, Hilden, Germany). One of the selective primers (CG, CHG, CHH) was γ^32^P-labeled using 5 U T4 polynucleotide kinase (NEB, US). The selective amplification DNA products were separated electrophoretically on a 7% polyacrylamide gel and exposed to X-ray films. The signals detected on X-ray films, reflecting fragments ranging from 50 to 700 base pairs, were converted into a binary 0–1 matrix with 1 (presence of DNA fragment) or 0 (absence of DNA fragment) for data analysis. The quantitative characteristics of TCIV and its components are given as percentages. The estimation method is based on the juxtaposition of DNA fragments obtained from digesting the DNA of the donor plant and its regenerant with two sets of restriction enzymes, Acc65I/MseI and KpnI/MseI. A 4-digit binary code is evaluated based on juxtaposed marker comparison. The first two digits reflect the presence (1) or absence (0) of a DNA fragment in the Acc65I/MseI matrix for the donor plant and the regenerant. The following two digits describe DNA fragments for the donor plant and the regenerant in the KpnI/MseI matrix. Sixteen such zero–one codes (0000 to 1111) are possible. Each code reflects a specific genetic background, i.e., the appearance of changes in the sequence or methylation of the DNA of the plants studied or the absence of any changes in the restriction sites and their surroundings recognized by the restriction enzymes used. Each code reflexed an “event” assigned to changes such as sequence variation (SV), de novo methylation (DNMV), or demethylation (DMV). Codes reflecting events of the same type were grouped, forming respective variation types after normalization by the total number of events. The exact formulas for converting the number of events into percentages are described in detail earlier (Machczyńska et al. 2014; Orłowska and Bednarek 2020). The selective primers with the -CHG and -CG sequences at the 3′ end reflect the symmetric context; those with any combination of A and T amplified the asymmetric CHH contexts (Table 1) (Orłowska and Bednarek 2020).

### Statistics

Linear regression analysis was conducted in IBM SPSS software v. 27 (IBMCorp 2021). The least absolute shrinkage and selection operator (LASSO) regression analysis was performed in XLSTSAT software (Addinsoft 2020) using the implemented GLMNET R package and predefined settings.

The structural equation modeling method (Jöreskog 1973; Keesling 1972; Kozak and Kang 2006; Wiley 1973) was used in the computer software IBM SPSS® AmosTM 20 (Arbuckle 2014). A theoretical model (postulated model) was verified on the basis of empirical data. The maximum likelihood (ML) estimation with the Levenberg–Marquardt iteration method (Levenberg 1944; Madsen et al. 2004; Marquardt 1963) was used.

## Results

The donor plants (24 individuals) did not differ in morphological characteristics (height, leaf size, tillering, or seed set) from the starting plants from which they were derived. Explants (anthers) were taken from all donor plants, but ultimately only one donor plant yielded regenerants under the eight in vitro culture conditions tested (A–H). These conditions differed in Cu(II) and Ag(I) ion concentration in the induction medium and the time for which anthers were incubated on the IM (Online Resource 1, Table S1). All in vitro culture conditions tested yielded 37 regenerants morphologically identical with the donor plants. In all treatments taken together, circa 49% of regenerated plants were albinotic. The number of regenerants in each trial varied and ranged from 3 to 10 (Online Resource 1, Table S1). Depending on the treatment, the green plant regeneration efficiency ranged from 0.87 green regenerants per 100 plated anthers in A to 6.06 green regenerants per 100 plated anthers in H (Online Resource 1, Table S1).

Thirteen pairs of selective primers were used to analyze metAFLP data for the donor plant and regenerants. A total of 597 markers were found, with 43 markers per primer expressing sequence context CG, 46 markers per primer typically representing sequence context CHG, and 51 markers representing asymmetric sequence context CHH. Using the Acc65I and MseI enzymes to digest DNA, metAFLP markers were analyzed, producing 574 bands. These markers serve as the 49.41% polymorphic loci and provide information on DNA methylation and sequence changes. As opposed to this, examining DNA markers cut with the KpnI and MseI enzymes produced 555 DNA markers representing 44.39% polymorphic loci. The results from KpnI and MseI enzyme cutting only talk about changes in the DNA sequence.

According to the metAFLP analysis based on markers for a donor plant and regenerants obtained in eight in vitro conditions, the mean value for sequence variation in the asymmetric CHH context (CHH_SV) was 8.65% (the complete data set is included in Online Resource 1, Table S1). Demethylation in the CHH sequence context (CHH_DMV) was 0.76%, while de novo DNA methylation in the CHH context (CHH_DNMV) was 0.58%. So, only data that was statistically significant and fit the SEM analysis’s assumptions were shown.

**Fig. 1:**
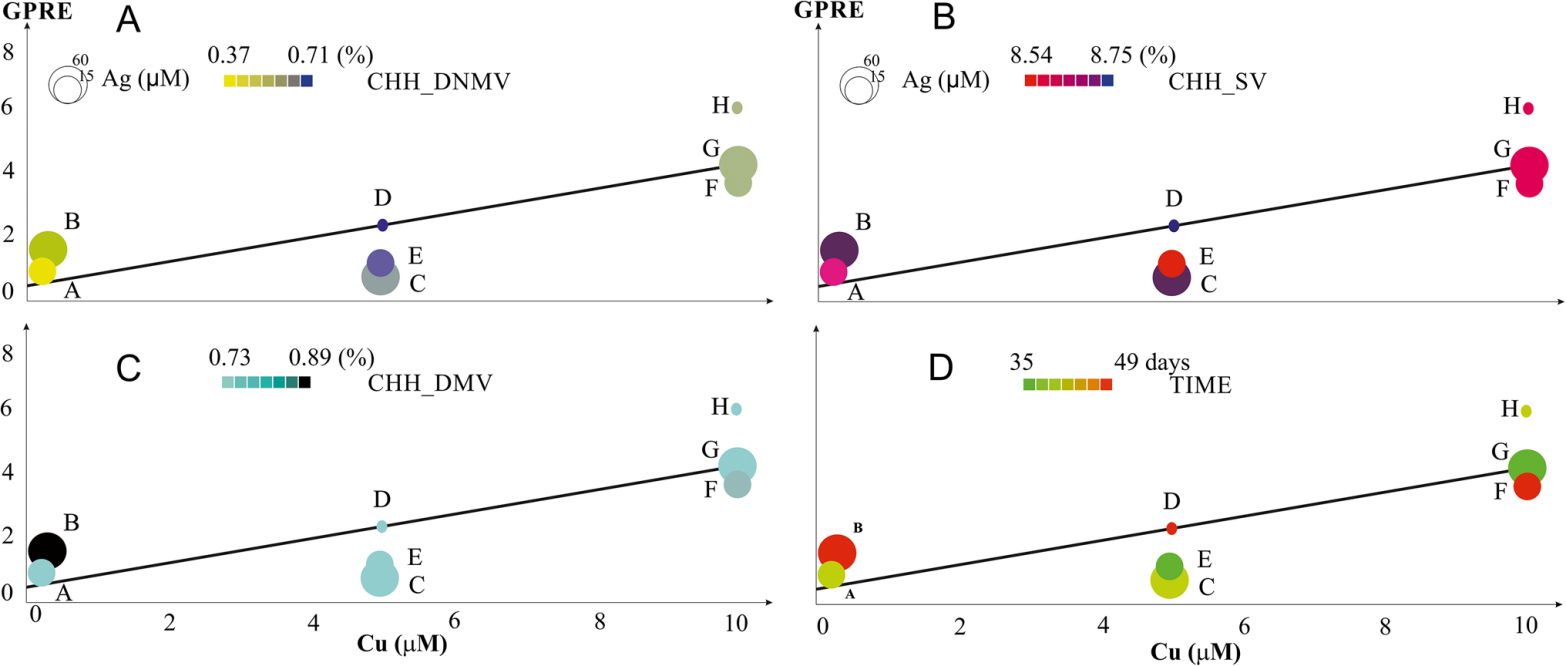
GPRE as a function of Cu(II) concentration in the IM illustrating the change in Cu(II), Ag(I) concentration, time of in vitro tissue culture and sequence variation, DNA demethylation, and changes in DNA de novo methylation, affecting asymmetric (CHH) sequence contexts. Axis *Y*, green plant regeneration efficiency (GPRE) reflecting the number of regenerants per 100 plated anthers; axis *X*, Cu(II) concentration (μM) in the IM. Ag(I) concentration (μM) in the IM are illustrated by the size of colored circles, whereas circle color corresponds to the level of metAFLP characteristics—de novo DNA methylation (CHH_DNMV) (**A**), sequence variation (CHH_SV) (**B**), and DNA demethylation (CHH_DMV) (**C**) between donor plant and its regenerants affecting asymmetric CHH sequence contexts; values expressed in percentages (%); time (days) of tissue cultures is given as colored circles (**D**); A–H reflects treatments; line is linear regression

Green plant regeneration efficiency increases with Cu(II) and decreases with Ag(I) ion concentration in the IM (Fig. 1). De novo DNA methylation (Fig. 1A) was the highest when Cu(II) reached a moderate concentration without Ag(I) (treatment D), and the lowest under low Cu(II) concentration and moderate Ag(I) (treatment A). Furthermore, low DNMV values were associated with low yields of green regenerants. Sequence variation (Fig. 1B) reached its highest value for treatment E, characterized by intermediate supplementation of both copper and silver. In contrast, the lowest SV was observed at intermediate Cu(II) levels and in the absence of Ag(I) supplementation. At the same time, extreme values of SV level were associated with intermediate production of green regenerants. Demethylation (Fig. 1C) reached values ranging from 0.73 to 0.89%, with the highest value observed at low Cu(II) supplementation and maximum Ag(I) level (treatment B). The lowest DMV was observed in two treatments (A and E) with low and intermediate Cu(II) and intermediate Ag(I) supplementation. Furthermore, the lowest yield of green regenerants was observed at the highest level of cytosine demethylation in the context of CHH. Also, the most regenerants were made when the anthers were left on IM for an average number of days (Fig. 1D).

The best GPRE was associated with intermediate levels of individual metAFLP characteristics. Analysis of the relationship between GPRE and Cu(II) concentration in IM using linear regression shows that as Cu(II) concentration went up, so did GPRE (GPRE = 0.3745 + 0.5237*Cu(II)). The determination coefficient equals 0.65.

Structural equation modeling was based on 37 samples shared between eight treatments. The essential variable characteristics used for the SEM analysis are in Table 2. Skewness and kurtosis were somewhat apart from null values, indicating deviation from the normal distribution. However, the analyzed variables were quantitative, and all met the conditions set out and the Lindeberg–Lévy theorem (Taboga 2012). So, it can be assumed that the distribution of these variables is asymptotically close to the theoretical normal distribution.

**Table 2:** Descriptive statistics of the variables present in the postulated models

Variable	Descriptive statistics			
	Mean	Variance	Skewness	Kurtosis
[Ag (I)]	22.432	713.363	0.704	− 1.480
[Cu (II)]	5.427	12.787	− 0.102	− 1.008
[CHH_DNMV]	0.584	0.019	− 0.194	− 1.054
[CHH_SV]	8.655	0.013	− 0.079	− 0.754
[GPRE]	2.556	2.752	0.932	− 0.122

Pearson’s linear correlation coefficients (Table 3) show that CHH_DNMV was negatively correlated with Ag(I) and positively correlated with Cu(II); CHH_SV was negatively correlated with Cu(II), whereas GPRE was positively correlated with Cu(II) and negatively correlated with CHH_SV.

**Table 3:** Pearson’s linear correlation coefficients for analyzed variables

Variable	[Ag(I)]	[Cu(II)]	[CHH_DNMV]	[CHH_SV]	[GPRE]
[Ag(I)]	1				
[Cu(II)]	− 0.181	1			
[CHH_DNMV]	− 0.426^**^	0.320^*^	1		
[CHH_SV]	0.05	− 0.386^**^	0.247	1	
[GPRE]	− 0.201	0.807^**^	0.005	− 0.297^*^	1

^*^Significant at *p* ≤ 0.05; ^**^significant at *p* ≤ 0.01

**Fig. 2:**
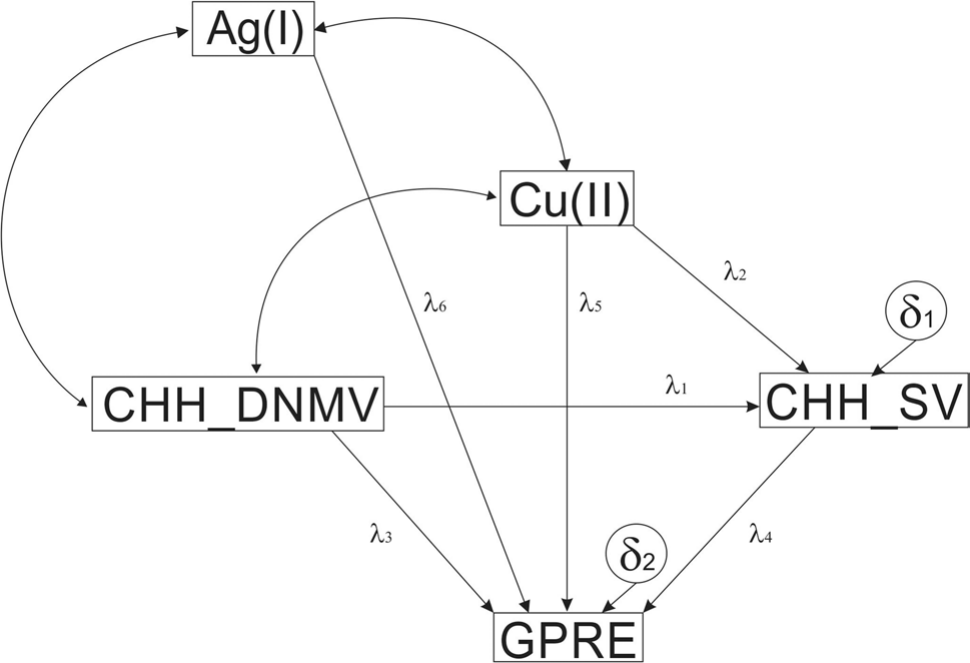
The path diagram of the postulated model. Ag (I), Cu(II), ion concentrations; GPRE, green plant regeneration efficiency; the metAFLP quantitative characteristics concerning sequence variation (CHH_SV) and de novo DNA methylation (CHH_DNMV) between donor plant and its regenerants affecting asymmetric CHH sequence contexts; *λ*_1_–*λ*_6_, path coefficients; *δ*_1_–*δ*_2_, residuals (experimental errors). Double arrows illustrate relationships between covariates

The postulated model encompassed three exogenous variables (CHH_DNMV, Cu (II), and Ag(I)). The remaining variables were endogenous. All relationships were non-recursive. There were three covariances in the model. All variables were observed. The model included two residuals (Fig. 2).

The chi-square statistics (Table 4) for the verification of fitting of the model are not statistically significant. A limited sample size (*n* = 37) may result in the acceptance of an incorrect model (Kenny and McCoach 2003); therefore, following MacCallum et al. (1996) recommendations, the general model fit test based on chi-square statistics was treated only as an information criterion. The model fit evaluation was based on the model’s descriptive goodness-of-fit measures.

**Table 4:** Summary of the analyzed structural equation model

Parameter	Postulated model
Degrees of freedom (*df*)	1
Chi-square (*χ*^2^)	1.2381
*p* value	0.2658
Root mean squares residuals (RMR)	0.3135
Standardized root mean squares residuals (SRMR)	0.0374
Goodness-of-fit index (GFI)	0.9888
Adjusted goodness-of-fit index (AGFI)	0.8316
Normed fit index (NFI)	0.9873
Relative fit index (RFI)	0.8729
Incremental fit index (IFI)	0.9975
Comparative fit index (CFI)	0.9979
Parsimonious normed fit index (PNFI)	0.0987
Parsimonious comparative fit index (PCFI)	0.0997
Root mean square error of approximation (RMSEA)	0.0744

The RMR represents the average residual value originating from fitting the variance–covariance (v–c) matrix for the postulated to the v–c matrix of the sample data. The residuals are relative to the size of the observed v–c and are challenging to interpret. The better index is the SRMR. It may range from 0 to 1. In well-fitting models, its value is assumed to be 0.05 or lower for excellent fitting (Hu and Bentler 1998, 1999), which is the case in the presented model.

The comparative indices of fit, namely GFI and AGFI, are based on comparing the postulated and standard models. The AGFI differs from the GFI in that it adjusts for the number of degrees of freedom in the model. Both indices could be classified as absolute indices as they compare the postulated model without a model. They range from 0 to 1.0, with values close to 1 indicating a good fit. The values of the postulated models were relatively high (0.9888 and 0.8316). They recommended that the greater they are, the better the model reflects relationships (Mulaik et al. 1989) which should be interpreted well-fitting the postulated model and sample data.

The NFI, CFI, and RFI indices are derived from the comparison of the postulated with the independence (null) model and provide a measure of complete covariation in the data and range from 0 to 1, with values close to 0.95 (Hu and Bentler 1998, 1999) indicating well-fitting. The NFI differs from the CFI in that it takes the sample size into account. Furthermore, the NFI may tend to underestimate fit in small samples. However, this is not the case in the given model as NFI and CFI statistics exceed the 0.95 cutoff value, whereas RFI is close to it, demonstrating that the model is well-fitting. The IFI index takes into account both the need for simplicity and the size of the sample. It is similar to the NFI, but it also takes into account the degrees of freedom, and a value close to 1 is in line with other fit statistics.

The parsimony-based indices of fit consider the number of estimated parameters of the postulated model in the assessment of overall model fit. The PNFI and PCFI indices were low, indicating that the postulated model is complex as the values were below the threshold level perceived as acceptable for other normed indices (James et al. 1982). As chi-square statistics were equal to 1.2381 and PNFI was low (0.0987), this indicates that the model is acceptable. The same reasoning is also valid for the PCFI fit statistics.

The RMSEA considers the error of approximation, and its value should be equal to or lower than 0.05 for excellent fitting or less than 0.08 for a suitable fitting model (Cudeck and Browne 1992; Jöreskog and Sörbom 1993). Therefore, its use is justified as it is sensitive to model misspecification and is conclusive to the model quality. Furthermore, the analysis demonstrates that the RMSEA value is shallow, supporting that the experimental data fits the postulated model.

The values of the coefficients for all paths in the postulated model, error variances, and covariances of the exogenous variables were estimated (Table 5). All path coefficients (*b*) were statistically significant.

**Table 5:** Path coefficients, variances, and covariances for the analyzed model

Parameter	Effect			Estimate (*b*)	Standard error	Test statistic	Standardized estimate (*β*)
*Path* *coefficients*							
*λ*_1_	[CHH_DNMV]	→	[CHH_SV]	0.3818	0.1113	9.4314^***^	0.5174
*λ*_2_	[Cu(II)]	→	[CHH_SV]	− 0.0156	0.0043	− 3.6421^***^	− 0.5438
*λ*_3_	[CHH_DNMV]	→	[GPRE]	− 5.0385	1.0668	− 4.7231^***^	− 0.4427
*λ*_4_	[CHH_SV]	→	[GPRE]	2.9840	1.2637	2.3614^*^	0.1954
*Λ*_5_	[Cu(II)]	→	[GPRE]	0.4792	0.0407	11.7636^***^	1.0914
*λ*_6_	[Ag(I)]	→	[GPRE]	− 0.0124	0.0045	− 2.7454^**^	− 0.1978
*Covariances*							
*φ*1	[CHH_DNMV]	↔	[Cu(II)]	0.2656	0.0407	2.9285^**^	0.4991
*φ*2	[Cu(II)]	↔	[Ag(I)]	− 0.8594	14.6886	− 0.0585	− 0.0089
*φ*3	[CHH_DNMV]	↔	[Ag(I)]	− 0.7380	0.5777	− 1.2775	− 0.1986
*Variances*							
*δ*_1_				0.0082	0.0018	4.6368^***^	
*δ*_2_				0.5474	0.5181	4.6368^***^	
[CHH_DNMV]				0.0205	0.0044	4.6368^***^	
[Cu(II)]				13.7940	2.9749	4.6368^***^	
[Ag(I)]				672.5904	145.0399	4.6368^***^	

^*^Significant at *p* ≤ 0.05; ^**^significant at *p* ≤ 0.01; ^***^significant at *p* ≤ 0.001

Based on standardized path coefficients (*β*) values of the postulated model, the most critical path was that describing the relationship between Cu(II) and GPRE, followed by the relationships between Cu(II) and CHH_SV, CHH_DNMV and CHH_SV, CHH_DNMV and GPRE, Ag(I) and GPRE, and ending with CHH_SV and GPRE. Only the covariance between Cu(II) and CHH_DNMV was significant.

The existing dependencies of the model can be broken down into direct, indirect, and total effects (Table 6). The GPRE variable showed the greatest dependence on Cu(II) (total *β* = 0.9852; including the direct effect as *β* = 0.1063, and the indirect effect as *β* = 1.0914). CHH_SV was most dependent on Cu(II) (= − 0.5438; direct and total effects).

**Table 6:** Direct, indirect, and total effects for the analyzed model

Effect			Estimates (*b*)			Standardized estimates (*β*)		
			Direct effect	Indirect effect	Total effect	Direct effect	Indirect effect	Total effect
[GPRE]								
[Ag(I)]	→	[GPRE]	− 0.0124	0	− 0.0124	− 0.1978	0	− 0.1978
[Cu(II)]	→	[GPRE]	0.4792	− 0.0466	0.4325	1.0914	0.1063	0.9852
[CHH_DNMV]	→	[GPRE]	− 5.0385	1.1392	− 3.8993	− 0.4427	1.1392	− 0.3426
[CHH_SV]	→	[GPRE]	2.984	0	2.984	0.1954	0	0.1954
[CHH_SV]								
[Ag(I)]	→	[CHH_SV]	0	0	0	0	0	0
[Cu(II)]	→	[CHH_SV]	− 0.0156	0	− 0.0156	− 0.5438	0	− 0.5438
[CHH_DNMV]	→	[CHH_SV]	0.3818	0	0.3818	0.5124	0	0.5124

GPRE is preferentially a function of CHH_DNMV, CHH_DMV, and Cu (II) based on LASSO regression, as indicated by coefficients corresponding to Optimal Lambda (determined by cross validation equaled 0.088964) of equation: GPRE = 0.200297 + 0.562864 CHH_DMV − 2.83904 CHH_DNMV + 0.399323 Cu(II) − 0.00469 Ag(I) + time 0.03484, whereas the other variables (CHH_SV, Ag(I), and time) appeared to be less important in determining GPRE.

## Discussion

Triticale is an important cereal crop in Europe and other regions of the world (Ayalew et al. 2018; Feledyn-Szewczyk et al. 2020). It is being exploited for animal feeding. However, several commercial triticale varieties, i.e., Hyt Prime, Hyt Max, and RGT KEAC, were evaluated (Geiger and Miedaner 2009), and its cytoplasmic male sterility system is being studied for hybrid breeding purposes (Wasiak et al. 2021). Its genotypic pool is limited due to the synthetic origin of the species (Wilson 1873). For breeding, it is best to use homozygous materials, which can be done through in vitro tissue cultures (androgenesis).

However, triticale regenerants are subjected to tissue culture-induced variation, which is rarely seen at the morphological level (Orłowska et al. 2022), with the exception of albino plants, which appear frequently (Krzewska et al. 2015; Orłowska et al. 2020). The presented data on the lack of morphological differences between a donor plant and regenerants is consistent with those published in the literature (Brettell et al. 1986; Oleszczuk and Banaszak 2016), where limited differences were noted. It was also observed that albino plants reached 49% of all regenerants, which is comparable to the data published by others (Krzewska et al. 2015). Thus, the presence of albinos is a factor limiting GPRE.

In the current study, the GPRE depended on the treatments and varied from 0.71 to 6.06, which is somewhat lower than the data available in the literature (from 2.48 to 20.88 green plantlets per 100 anthers) (Lantos et al. 2014). Furthermore, the regenerants originated from a single DH plant, whereas elevated values were for different genotypes with distinct androgenic responses (Lantos et al. 2014). Moreover, the presence of Cu(II) and Ag(I) in the IM should not be neglected.

The metAFLP analysis, which aimed at estimating the level of in vitro*-*induced variation and its components, showed that average de novo methylation, sequence variation, and DNA demethylation levels were different for the treatments (Online Resource 1, Table S1). Furthermore, the level of DNA sequence changes in the asymmetric CHH context was higher than that of DNA demethylation (11 × higher) or de novo DNA methylation (15 × higher). The presented data agrees with others (Machczyńska et al. 2015), where a higher sequence variation was observed in triticale regenerants derived from anther without focusing on a specific sequence context, isolated microspore, and immature zygotic embryo cultures. Analysis of DNA methylation changes in triticale in the present study showed that demethylation (0.76%) exceeded de novo methylation (0.58%). In contrast, similar estimations made for anther culture-derived barley regenerants displayed that DMV and DNMV take values close to each other (0.08% each) (Orłowska and Bednarek 2020), but tenfold lower than presented here for triticale. The low level of methylation changes in the CHH context reflects the low methylation of the region compared to all other contexts, as indicated in Arabidopsis, where 22% for CG, 5.9% for CHG, and 1.5% for CHH were methylated. Similar data in rice states that 59% for CG, 21% for CHG, and 2.2% for CHH (Feng et al. 2010) contexts are methylated. However, the same figures in maize are as follows: 86% for CG, 74% for CHG, and 5.4% for CHH (Gent et al. 2013). Furthermore, the methylation context of CHH is also the least represented of all three contexts in the repetitive regions of the plant genome (Feng et al. 2010; Zemach et al. 2010), which are more than 85% in wheat (Brenchley et al. 2012; Zimin et al. 2017) and 90% in rye (Bartoš et al. 2008; Flavell et al. 1974). The explanation of the lowered CHH sequence context methylation may also reflect varying mechanisms and pathways of methylation maintenance in symmetric and asymmetric contexts (Law and Jacobsen 2010). The CHH methylation is maintained by epigenetic mechanisms involving RNA-directed DNA methylation (RdDM) (Chakraborty et al. 2021; Matzke and Mosher 2014). Moreover, Cu(II) and Ag(I) ingredients may affect biochemical pathways influencing TCIV and GPRE. It is enough to mention that Cu(II) and Ag(I) may promote the callus induction (AboShama and Atwa 2019; Malik et al. 2021). Also, both ions stimulate green plant regeneration (Makowska et al. 2017; Mohiuddin et al. 1997; Nirwan and Kothari 2003). The way AgNO_3_ affects the GPRE is not apparent. One option is that silver acts as an inhibitor of ethylene receptors, blocking ethylene’s negative effects on developing tissue cultures (Jha et al. 2007). Another explanation is that the Ag(I) and Cu(II) ions have similar physicochemical properties (Roe et al. 1990). According to this hypothesis, Ag(I) may replace Cu in, for example, Cu/Zn-SOD (Ciriolo et al. 1994), disrupting ROS scavenging, affecting methylated cytosines, and causing DNA mutations due to oxidative stress (Dutta et al. 2018). Therefore, it is supposed that Ag(I) can displace Cu(II) from various complexes and thus affects GPRE. However, the postulated model does not support such a notion as the path between Cu(II) and Ag(I) and the covariance between the variables was insignificant while the paths towards GPRE were. Furthermore, the effect of Ag(I) on GPRE is surprisingly negative, contradicting the notion that it has a positive effect on GPRE. Thus, the Ag(I) action mechanism is unknown; however, according to the proposed model, Cu(II) may affect GPRE via cytosine methylation and can induce mutations via, for example, oxidative processes, influencing the productivity of green regenerants. Whatever the nature of Cu(II) action on GPRE, as indicated by the model, CHH_SV influences GPRE, which seems to be the result of Cu(II) ions.

In general, increasing Cu(II) concentration led to changes in CHH_SV, CHH_DNMV, and CHH_DMV, resulting in an increase in the GPRE (Fig. 1). It should be stressed that the highest values of GPRE were obtained under the highest Cu(II) concentrations and a lack of Ag(I) in the IM. At the same time, CHH_SV, CHH_DMV, CHH_DNMV, and time were at medium levels. Therefore, when the highest Cu(II) and Ag(I) concentrations were applied, the GPRE decreased. Due to the limits of the experiment, only the average GPRE for each treatment could be measured. Since regenerants from each treatment came from the same donor plant, statistics could not tell if the difference between conditions with and without Ag(I) was significant.

The Cu(II) concentrations used in the experiment were below 20 mg l^−1^, which is assumed to be toxic (Nawrot-Chorabik 2017). Thus, I tend to think that Cu(II) at the highest concentrations (10 mM [2.5 mg l^−1^], which is still below the toxic level) stabilized the biochemical pathways (ETC, Yang cycle) as DNA methylation and demethylation levels were kept at intermediate levels compared to the other experimental conditions. Furthermore, the intermediate level of SV affecting CHH sequences could be interpreted as proper Cu/Zn-SOD functioning, reducing ROS levels. The notion seems to be supported because the GPRE decreased when Ag(I) in the IM was at its highest. Under such conditions, it could be expected that Ag(I) could substitute Cu(II) in Cu/Zn-SOD, resulting in increased CHH_SV and decreased CHH_DNMV. Still, it was not the case, suggesting that other factors should be involved here. The putative candidates are melatonin or GSH, which may act as ROS scavengers. Unfortunately, no evidence supports this hypothesis, and more tests are needed to support it.

It is worth mentioning that increasing Cu(II) concentration led to better GPRE as indicated by linear regression analysis and fits the concept of the stabilizing effect of Cu(II) on cell functioning for the GPRE. This may indicate that, at least in triticale, Cu(II) is a crucial factor influencing GPRE. Interestingly, however, LASSO analysis showed that CHH_DNMV should negatively control the GPRE. GPRE is also controlled by CHH_DMV and Cu(II) concentration but to a lesser extent. Therefore, the role of Ag(I) in the GPRE seems to be limited. The latter is not congruent with results on barley, where silver ions seemed to be essential for the GPRE (Bednarek and Orlowska 2020). Possibly, such a discrepancy is due to the action of Ag(I) on ETR1 receptors of ethylene. So, Ag(I) ions may stimulate callus development, as has been suggested before (Shah et al. 2014; Wu et al. 2006), but their effects on plant regeneration depend on the cultivar (Zang et al. 2001). Unfortunately, it cannot be ruled out that other Ag(I)-dependent mechanisms exist.

Structural equation modeling is a statistical tool based on the maximum likelihood method to construct models explaining complex phenomena. As LASSO indicated that CHH_DNMV, CHH_DMV, Cu(II), and Ag(I) might influence the GPRE, and considering that sequence variation may also affect gene functioning, all the variables were implemented in the model. The analyzed fit statistics showed that the model was stable and capable of demonstrating relationships between variables where all paths were significant. It should be noted that Cu(II) most likely reflects the ETC, gene expression regulated by the Yang cycle and its outcomes, and the combined effects of Cu/Zn-SOD, GSH, and melatonin on the GPRE. The Cu(II) is at the center of the model and is the main factor that determines the GPRE. This is shown by the fact that the Cu(II) → GPRE path has the highest direct effect value. Evidently, Cu(II) positively influences the GPRE.

On the other hand, Cu(II) negatively affects the CHH_SV. Therefore, it could be expected that the level of oxidative stress should increase with increasing Cu(II) concentration. However, this seems not to be the case, at least at the level of averaged effects, because GSH and melatonin could remove the increased level of ROS or other ROS scavengers, whose synthesis may also increase with Cu(II), explaining the negative value of the Cu(II) → CHH_SV path. Remarkably, the CHH_DNMV → GPRE path is also negative, suggesting that de novo methylation of the CHH contexts may limit the GPRE if the indirect effect is omitted.

Keeping in mind that de novo methylation may limit the expression of genes responsible for the GPRE, the negative value of the path is apparent. Still, when the indirect effect of the CHH_DNMV → GPRE path is analyzed, the *b* value is the highest, indicating its highest input to GPRE. The CHH_DNMV → CHH_SV path seems to reflect oxidative stress leading to sequence variation. SEM shows that stress plays an essential role in explaining the level of CHH_SV, while CHH_SV only slightly affects the GPRE. In the CHH_SV → GPRE path, the *β* value is the smallest but positive, suggesting SV (mutations) influences the GPRE. Although not presented here (could be delivered on demand), there was also verified an alternative model where Cu(II) directly influenced CHH_DNMV, which might illustrate oxidative stress’s role in CHH_DNMV. The path was significant, and the direct effect was relatively large. However, some of the model fit indices were slightly lower than those discussed here. Thus, it cannot be ruled out that the Cu(II) → CHH_DNMV pathway will need to be modified when additional variables are introduced into the model.

The Ag(I) is included in the model. It exhibits only a direct effect on GPRE without influencing other variables. Furthermore, its effect is relatively small and negative, which should be interpreted as undesirable in terms of GPRE. The results show that Ag(I) ions cannot replace Cu(II) ions as cofactors in enzymatic reactions. The notion agrees that Ag(I) correlates poorly with Cu(II). The result contrasts with suggestions presented by the others (Kumar et al. 2009), suggesting the opportunity for substitution. Alternatively, the Ag(I) concentration in the IM was too low to reveal the action of silver ions on Cu(II). There were tests on whether Ag(I) influences CHH_DNMV, but the respective SEM model (which could be supplemented if requested) was not acceptable for model fit indices. So, it is not clear how Ag(I) works, and it is not known if Ag(I) affects GPRE because it is linked to the ETR1 receptor. However, if Ag(I) substitutes Cu(II) in the ETR1 receptor, the ethylene action might be disturbed (Hassan and Islam 2021), resulting in the decrease of GPRE. Thus, some epigenetic mechanisms involving the ETR1 receptor cannot be excluded. Furthermore, Ag(I) may have an effect on the efficiency of callus formation (Wu et al. 2006). Whatever the mechanism, the data show that Ag(I) in the IM is not a good choice for GPRE in triticale, which contradicts previous findings (Harathi and Naidu 2016 7086).

The linear regression model confirms that with increasing Cu(II) concentration, the GPRE increases, whereas LASSO shows that many factors may participate in the GPRE. Finally, the SEM model demonstrates the central role of Cu(II) in the IM for the GPRE and the involvement of many biochemical pathways and cellular mechanisms in the GPRE. There was a limited impact of silver ions in IM and CHH_SV variations on GPRE compared to Cu(II). It was also not possible to demonstrate an effect of anther culture time on any type of variation implemented in the model or on GPRE.

## Supplementary Information

Below is the link to the electronic supplementary material.

Supplementary file1 (DOCX 16 KB)

## Data Availability

All data generated during this study are included in this published article.
