# Supplementary material for: Triticale doubled haploid plant regeneration factors linked by structural equation modeling
Source: J Appl Genet. 2022 Aug 26;63(4):677–90. doi: 10.1007/s13353-022-00719-7 (PMC9637073; doi:10.1007/s13353-022-00719-7)
Supplement: Supplementary file 1 — Supplementary file1 (DOCX 16 KB) [file 13353_2022_719_MOESM1_ESM.docx]

**Title:** Triticale doubled haploid plant regeneration factors linked by structural equation modeling

**Journal:** Journal of Applied Genetics

**Author:** Renata Orłowska^1*^

^1^Plant Breeding and Acclimatization Institute – National Research Institute, 05-870 Błonie, Radzików,

^*^Corresponding author: r.orlowska@ihar.edu.pl, ORCID: https://orcid.org/0000-0001-7150-6920

**Online Resource 1**

**Table S1** The arrangement of *in vitro* tissue culture conditions, including Cu(II) and Ag(I) ion concentrations in the induction medium (IM), time (days) of anther culture, efficiency of green plant regeneration (GPRE, expressed as the number of green regenerants per 100 plated anthers) contraposed with treatments (A-H), and the metAFLP quantitative characteristics concerning asymmetric CHH sequence contexts

| Treatment | *In vitro* anther culture conditions | | | metAFLP quantitative characteristics (%) | | | GPRE |
| --- | --- | --- | --- | --- | --- | --- | --- |
|  | Cu (µM) | Ag (µM) | time (days) | CHH_SV | CHH_DMV | CHH_DNMV |  |
| A | 0.1 | 10 | 42 | 8.66 | 0.74 | 0.37 | 0.87 |
| A | 0.1 | 10 | 42 | 8.66 | 0.74 | 0.37 | 0.87 |
| A | 0.1 | 10 | 42 | 8.52 | 0.73 | 0.36 | 0.87 |
| B | 0.1 | 60 | 49 | 8.64 | 0.92 | 0.37 | 1.52 |
| B | 0.1 | 60 | 49 | 8.64 | 0.92 | 0.37 | 1.52 |
| B | 0.1 | 60 | 49 | 8.79 | 0.93 | 0.37 | 1.52 |
| B | 0.1 | 60 | 49 | 8.79 | 0.93 | 0.37 | 1.52 |
| B | 0.1 | 60 | 49 | 8.79 | 0.75 | 0.56 | 1.52 |
| C | 5 | 60 | 42 | 8.76 | 0.75 | 0.75 | 0.71 |
| C | 5 | 60 | 42 | 8.79 | 0.75 | 0.56 | 0.71 |
| C | 5 | 60 | 42 | 8.64 | 0.73 | 0.55 | 0.71 |
| D | 5 | 0 | 49 | 8.64 | 0.73 | 0.55 | 2.38 |
| D | 5 | 0 | 49 | 8.64 | 0.73 | 0.55 | 2.38 |
| D | 5 | 0 | 49 | 8.76 | 0.75 | 0.75 | 2.38 |
| D | 5 | 0 | 49 | 8.76 | 0.75 | 0.75 | 2.38 |
| D | 5 | 0 | 49 | 8.76 | 0.75 | 0.75 | 2.38 |
| D | 5 | 0 | 49 | 8.76 | 0.75 | 0.75 | 2.38 |
| D | 5 | 0 | 49 | 8.76 | 0.75 | 0.75 | 2.38 |
| D | 5 | 0 | 49 | 8.76 | 0.75 | 0.75 | 2.38 |
| D | 5 | 0 | 49 | 8.76 | 0.75 | 0.75 | 2.38 |
| D | 5 | 0 | 49 | 8.91 | 0.76 | 0.76 | 2.38 |
| E | 5 | 10 | 35 | 8.63 | 0.73 | 0.73 | 1.17 |
| E | 5 | 10 | 35 | 8.63 | 0.73 | 0.73 | 1.17 |
| E | 5 | 10 | 35 | 8.48 | 0.72 | 0.72 | 1.17 |
| E | 5 | 10 | 35 | 8.48 | 0.72 | 0.72 | 1.17 |
| E | 5 | 10 | 35 | 8.50 | 0.72 | 0.54 | 1.17 |
| F | 10 | 10 | 49 | 8.48 | 0.90 | 0.54 | 3.79 |
| F | 10 | 10 | 49 | 8.65 | 0.74 | 0.55 | 3.79 |
| F | 10 | 10 | 49 | 8.65 | 0.74 | 0.55 | 3.79 |
| G | 10 | 60 | 35 | 8.62 | 0.75 | 0.56 | 4.24 |
| G | 10 | 60 | 35 | 8.49 | 0.74 | 0.55 | 4.24 |
| G | 10 | 60 | 35 | 8.49 | 0.74 | 0.55 | 4.24 |
| G | 10 | 60 | 35 | 8.65 | 0.74 | 0.55 | 4.24 |
| H | 10 | 0 | 42 | 8.49 | 0.74 | 0.55 | 6.06 |
| H | 10 | 0 | 42 | 8.49 | 0.74 | 0.55 | 6.06 |
| H | 10 | 0 | 42 | 8.65 | 0.74 | 0.55 | 6.06 |
| H | 10 | 0 | 42 | 8.65 | 0.74 | 0.55 | 6.06 |
